# Supplementary figures and images for: Improving insect conservation across heterogeneous landscapes using species–habitat networks
Source: PeerJ. 2021 Jan 5;9:e10563. doi: 10.7717/peerj.10563 (PMC7792512; doi:10.7717/peerj.10563)

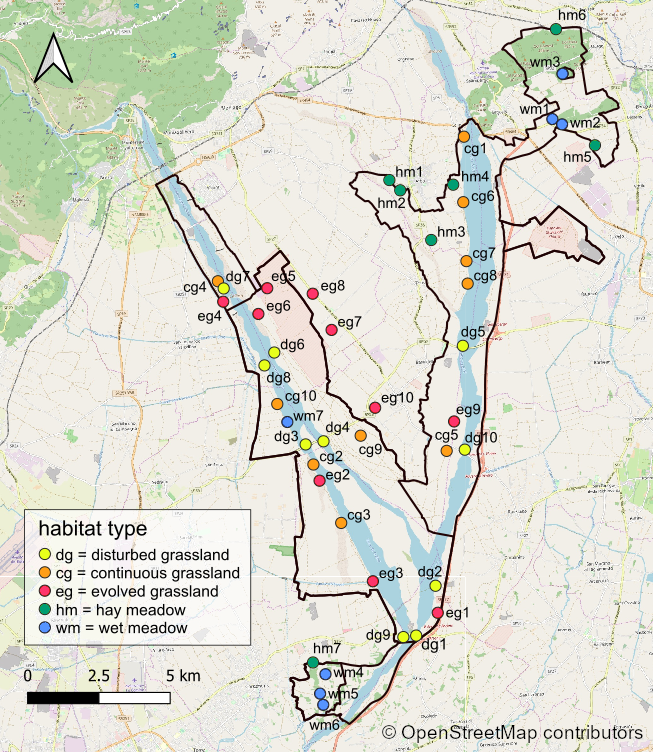

Supplement: Supplemental Information 1 — Map of the 44 sampling sites within the Special Protection Area “Magredi di Pordenone” (SPA-IT 33110011). Map credit: ©OpenStreetMap contributors. [file peerj-09-10563-s001.png]

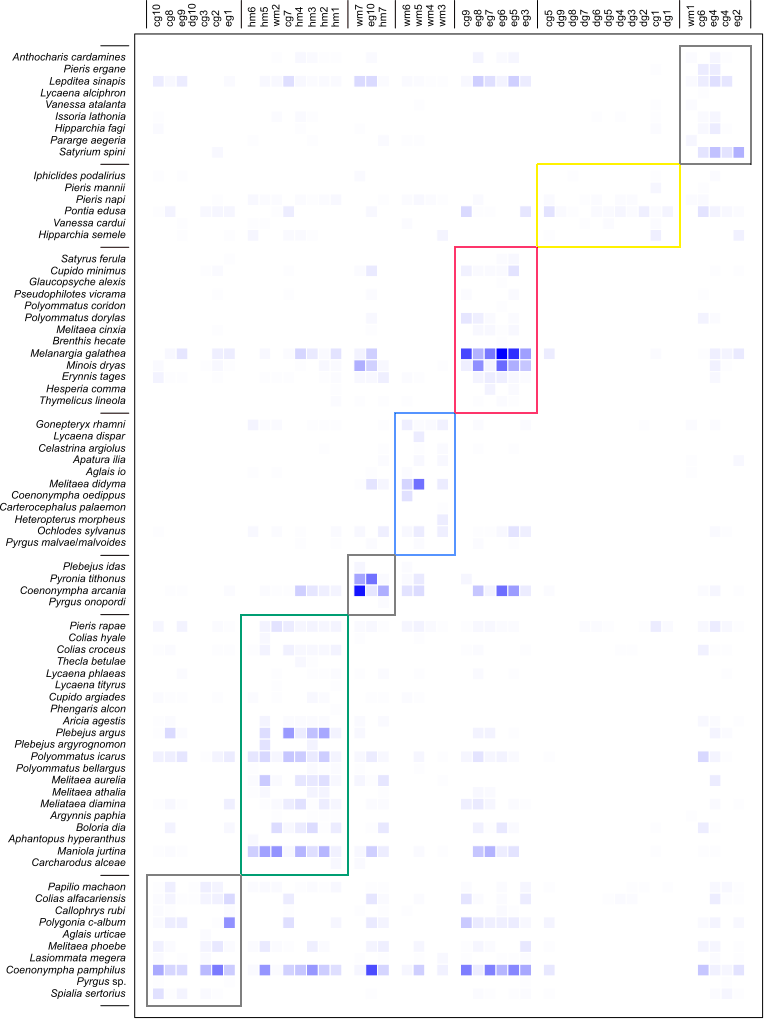

Supplement: Supplemental Information 2 [file peerj-09-10563-s002.png]
