# Supplementary material for: Improving insect conservation across heterogeneous landscapes using species–habitat networks
Source: PeerJ. 2021 Jan 5;9:e10563. doi: 10.7717/peerj.10563 (PMC7792512; doi:10.7717/peerj.10563)
Supplement: Supplemental Information 3 — Number of sampling patches, management strategy, level of disturbance, bare ground cover, and shrub cover for each habitat type. * indicates habitat types included in the list of Natura 2000 protected habitats. [file peerj-09-10563-s003.docx]

|  | Disturbed  grassland | Continuous  grassland* | Evolved  grassland* | Hay  meadow | Wet  meadow |
| --- | --- | --- | --- | --- | --- |
| Number of patches | 10 | 10 | 10 | 7 | 7 |
| Management | Un-managed | Un-managed | Un-managed | Mown twice a year | Mown once  every 1-2 years |
| Fertilization | No | No | No | <50 kg N ha^-1^ yr^-1^ | No |
| Soil disturbance by flood | Recent | Medium | Undisturbed | None | None |
| Bare ground cover | > 75% | 10-30% | <10% | 0% | 0% |
| Shrub cover | < 5% | < 5% | > 15% | 0% | 0% |
| Soil water conditions | Extremely dry | Dry | Dry | Mesic | Wet |
