# Supplementary material for: Improving insect conservation across heterogeneous landscapes using species–habitat networks
Source: PeerJ. 2021 Jan 5;9:e10563. doi: 10.7717/peerj.10563 (PMC7792512; doi:10.7717/peerj.10563)
Supplement: Supplemental Information 4 — List of sampled butterfly families and species with total abundance and frequency in the 44 sampling sites. * indicates protected and vulnerable butterfly species. [file peerj-09-10563-s004.docx]

| **Family** | **Species** | **Abundance** | **Frequency** |
| --- | --- | --- | --- |
| **Hesperiidae** |  | **251** | **28** |
|  | *Carcharodus alceae* | 7 | 2 |
|  | *Carterocephalus palaemon* | 1 | 1 |
|  | *Erynnis tages* | 71 | 20 |
|  | *Hesperia comma* | 11 | 3 |
|  | *Heteropterus morpheus* | 10 | 1 |
|  | *Ochlodes sylvanus* | 83 | 17 |
|  | *Pyrgus malvae*/*malvoides* | 9 | 6 |
|  | *Pyrgus onopordi* | 2 | 1 |
|  | *Pyrgus* sp. | 4 | 4 |
|  | *Spialia sertorius* | 44 | 14 |
|  | *Thymelicus lineola* | 9 | 7 |
| **Lycaenidae** |  | **915** | **31** |
|  | *Aricia agestis* | 30 | 12 |
|  | *Callophrys rubi* | 13 | 9 |
|  | *Celastrina argiolus* | 8 | 6 |
|  | *Glaucopsyche alexis* | 1 | 1 |
|  | *Lycaena alciphron* | 1 | 1 |
|  | *Lycaena dispar** | 11 | 1 |
|  | *Lycaena phlaeas* | 16 | 11 |
|  | *Lycaena tityrus* | 2 | 2 |
|  | *Phengaris alcon** | 1 | 1 |
|  | *Plebejus argus* | 229 | 15 |
|  | *Plebejus argyrognomon* | 23 | 2 |
|  | *Plebejus idas* | 7 | 2 |
|  | *Polyommatus bellargus* | 222 | 19 |
|  | *Polyommatus coridon* | 1 | 1 |
|  | *Polyommatus dorylas* | 41 | 7 |
|  | *Polyommatus icarus* | 287 | 28 |
|  | *Pseudophilotes vicrama* | 10 | 6 |
|  | *Satyrium spini* | 7 | 3 |
|  | *Thecla betulae** | 5 | 2 |
| **Nymphalidae** |  | **4061** | **37** |
|  | *Aglais io* | 6 | 5 |
|  | *Aglais urticae* | 2 | 1 |
|  | *Apatura ilia* | 15 | 5 |
|  | *Aphantopus hyperantus* | 4 | 1 |
|  | *Argynnis paphia* | 5 | 5 |
|  | *Boloria dia* | 129 | 17 |
|  | *Brenthis hecate* | 1 | 1 |
|  | *Coenonympha arcania* | 491 | 24 |
|  | *Coenonympha oedippus** | 17 | 1 |
|  | *Coenonympha pamphilus* | 1022 | 31 |
|  | *Hipparchia fagi* | 23 | 7 |
|  | *Hipparchia semele* | 38 | 10 |
|  | *Issoria lathonia* | 18 | 9 |
|  | *Lasiommata megera* | 22 | 13 |
|  | *Maniola jurtina* | 407 | 23 |
|  | *Melanargia galathea* | 711 | 24 |
|  | *Melitaea athalia* | 116 | 12 |
|  | *Melitaea aurelia* | 25 | 7 |
|  | *Melitaea cinxia* | 15 | 6 |
|  | *Melitaea diamina* | 137 | 6 |
|  | *Melitaea didyma* | 99 | 20 |
|  | *Melitaea phoebe* | 113 | 22 |
|  | *Minois dryas* | 342 | 19 |
|  | *Pararge aegeria* | 12 | 6 |
|  | *Polygonia c-album* | 9 | 6 |
|  | *Pyronia tithonus* | 154 | 6 |
|  | *Satyrus ferula* | 114 | 5 |
|  | *Vanessa atalanta* | 4 | 3 |
|  | *Vanessa cardui* | 10 | 7 |
| **Papilionidae** |  | **75** | **28** |
|  | *Iphiclides podalirius* | 9 | 6 |
|  | *Papilio machaon* | 66 | 24 |
| **Pieridae** |  | **971** | **44** |
|  | *Anthocharis cardamines* | 35 | 12 |
|  | *Colias alfacariensis* | 123 | 21 |
|  | *Colias croceus* | 56 | 19 |
|  | *Colias hyale* | 6 | 3 |
|  | *Cupido argiades* | 19 | 12 |
|  | *Cupido minimus* | 54 | 12 |
|  | *Gonepteryx rhamni* | 46 | 16 |
|  | *Leptidea sinapis* | 257 | 28 |
|  | *Pieris ergane* | 22 | 3 |
|  | *Pieris mannii* | 6 | 2 |
|  | *Pieris napi* | 46 | 21 |
|  | *Pieris rapae* | 148 | 33 |
|  | *Pontia edusa* | 153 | 26 |
