# Supplementary material for: Improving insect conservation across heterogeneous landscapes using species–habitat networks
Source: PeerJ. 2021 Jan 5;9:e10563. doi: 10.7717/peerj.10563 (PMC7792512; doi:10.7717/peerj.10563)
Supplement: Supplemental Information 5 [file peerj-09-10563-s005.docx]

| **Habitat type** | **Patch code** | **Butterfly abundance** | **Butterfly richness** |
| --- | --- | --- | --- |
| **Disturbed grassland** |  | **68** | **10** |
|  | pg3 | 8 | 5 |
|  | pg5 | 12 | 5 |
|  | pg2 | 12 | 4 |
|  | pg5 | 7 | 4 |
|  | pg6 | 8 | 4 |
|  | pg1 | 10 | 3 |
|  | pg7 | 4 | 3 |
|  | pg8 | 2 | 2 |
|  | pg19 | 2 | 2 |
|  | pg9 | 3 | 1 |
| **Continuous grassland** |  | **1324** | **44** |
|  | ig10 | 133 | 25 |
|  | ig6 | 165 | 22 |
|  | ig7 | 222 | 21 |
|  | ig8 | 117 | 20 |
|  | ig2 | 143 | 19 |
|  | ig4 | 69 | 18 |
|  | ig9 | 305 | 18 |
|  | ig1 | 39 | 13 |
|  | ig3 | 75 | 11 |
|  | ig5 | 56 | 11 |
| **Evolved grassland** |  | **2655** | **54** |
|  | eg4 | 206 | 32 |
|  | eg8 | 322 | 30 |
|  | eg5 | 417 | 28 |
|  | eg10 | 398 | 28 |
|  | eg6 | 435 | 24 |
|  | eg7 | 288 | 22 |
|  | eg9 | 99 | 19 |
|  | eg1 | 166 | 18 |
|  | eg3 | 224 | 18 |
|  | eg2 | 100 | 17 |
| **Hay meadow** |  | **1368** | **51** |
|  | hm7 | 185 | 31 |
|  | hm1 | 135 | 29 |
|  | hm3 | 240 | 28 |
|  | hm2 | 225 | 26 |
|  | hm4 | 242 | 23 |
|  | hm5 | 260 | 21 |
|  | hm6 | 81 | 18 |
| **Wet meadow** |  | **858** | **51** |
|  | wm7 | 314 | 26 |
|  | wm5 | 207 | 25 |
|  | wm1 | 137 | 23 |
|  | wm6 | 101 | 18 |
|  | wm3 | 71 | 17 |
|  | wm1 | 19 | 9 |
|  | wm4 | 9 | 4 |
| **Total** |  | **6273** | **74** |
